# RAC3 Inhibition Induces Autophagy to Impair Metastasis in Bladder Cancer Cells *via* the PI3K/AKT/mTOR Pathway

**DOI:** 10.3389/fonc.2022.915240

**Published:** 2022-06-30

**Authors:** Liwei Wang, Jiazhong Shi, Sha Liu, Yaqin Huang, Hua Ding, Baixiong Zhao, Yuting Liu, Wuxing Wang, Jin Yang, Zhiwen Chen

**Affiliations:** ^1^ Urology Institute of People’s Liberation Army, Southwest Hospital, Third Military Medical University (Army Medical University), Chongqing, China; ^2^ Unit 32357 of People’s Liberation Army, Pujiang, China; ^3^ Department of Cell Biology, Third Military Medical University (Army Medical University), Chongqing, China

**Keywords:** bladder cancer, RAC3, autophagy, metastasis, PI3K/AKT/mTOR

## Abstract

**Background:**

Bladder cancer (BCa) is one of the most frequent malignant tumors globally, with a significant morbidity and mortality rate. Gene expression dysregulation has been proven to play a critical role in tumorigenesis. Ras-related C3 botulinum toxin substrate3 (RAC3), which is overexpressed in several malignancies and promotes tumor progression, has been identified as an oncogene. However, RAC3 has important but not fully understood biological functions in cancer. Our research aims to reveal the new functions and potential mechanisms of RAC3 involved in BCa progression.

**Methods:**

We explored the expression level of RAC3 and its relationship with prognosis by publicly accessible BCa datasets, while the correlation of RAC3 expression with clinicopathological variables of patients was analyzed. *In vitro* and *in vivo* proliferation, migration, autophagy, and other phenotypic changes were examined by constructing knockdown(KD)/overexpression(OE) RAC3 cells and their association with PI3K/AKT/mTOR pathway was explored by adding autophagy-related compounds.

**Results:**

Compared with non-tumor samples, RAC3 was highly expressed in BCa and negatively correlated with prognosis. KD/OE RAC3 inhibited/promoted the proliferation and migration of BCa cells. Knockdown RAC3 caused cell cycle arrest and decreased adhesion without affecting apoptosis. Inhibition of RAC3 activates PI3K/AKT/mTOR mediated autophagy and inhibits proliferation and migration of BCa cells *in vivo* and *in vitro*. Autophagy inhibitor 3MA can partially rescue the metastasis and proliferation inhibition effect caused by RAC3 inhibition. Inhibit/activate mTOR enhanced/impaired autophagy, resulting in shRAC3-mediated migration defect exacerbated/rescued.

**Conclusion:**

RAC3 is highly expressed in BCa. It is associated with advanced clinicopathological variables and poor prognosis. Knockdown RAC3 exerts an antitumor effect by enhancing PI3K/AKT/mTOR mediated autophagy. Targeting RAC3 and autophagy simultaneously is a potential therapeutic strategy for inhibiting BCa progression and prolonging survival.

## Introduction

BCa is one of the most common cancers worldwide, with 573278 new cases and 212536 deaths reported in 2020 worldwide (1). The incidence is higher in Western societies (2), with more than 81000 new cases and 17000 deaths annually in the United States alone (3). NMIBC (non-muscle invasive bladder cancer) accounts for nearly 70% of newly diagnosed BCa, whereas MIBC (muscle-invasive bladder cancer) and metastatic disease account for the remaining 30% (2, 4). Around 15% to 20% of NMIBC patients develop MIBC, which is more likely to migrate to lymph nodes and other organs (5). Lymph node metastasis will be found in about 25% of MIBC and 8% of high-risk NMIBC patients at the time of implementing radical cystectomy. Patients with this type of cancer usually have a dismal prognosis, with a relative 5-year OS of 15% (6). Surgery, radiotherapy, chemotherapy, and immunotherapy are the primary treatment modalities for BCa patients. Unfortunately, their efficacy is limited, and as a result, clinical outcomes remain far from ideal. The large-scale cancer sequencing efforts promote the advances of targeted therapy (7), help to find and study the core genes leading to the occurrence and development of BCa, and are of great significance in guiding the targeted therapy and prognosis of BCa. As members of the Ras superfamily, Rho GTPases are essential regulators of cellular function (8). Neoplastic biological activities involving Rho GTPase are indispensable for the progression of malignancies, including BCa (9). RAC is a member of the Rho GTPase subfamily (10), including three isoforms of RAC1, RAC2, and RAC3, and their homology exceeds 90% (11). There is substantial evidence that RAC proteins are involved in various cellular processes (12–15). Among them, RAC3 is overexpressed and is involved in the initiation and progression of different types of cancers, including brain tumors (16), lung cancer (17), breast cancer (18–21), prostate cancer (22), esophageal cancer (23) and ovarian cancer (24). There are several studies of RAC3 in BCa, including bioinformatics analysis based on sequencing data from publicly available databases (25–29), analysis based on the HPA database (29) and surgically derived BCa tissue samples (30), and also including findings of cell proliferation and migration phenotypes (31). However, a more comprehensive *in vitro* and *in vivo* experiment exploring how RAC3 contributes to BCa progression and its mechanism is still lacking.

Cancer metastasis is the leading cause of cancer-related death, and many lines of evidence indicate that autophagy is closely related to cancer metastasis (32–34). There is controversy in the study of the autophagy process in tumors, either inhibiting cancer cell survival or promoting cell proliferation (35–38). Autophagy is a multistep lysosomal degradation process that degrades and recycles proteins and organelles to maintain cellular homeostasis, a process that is important in a broad array of both physiological and pathological processes, including tumor growth. Multiple gene-mediated changes in autophagy flux are associated with cell proliferation and migration alterations, which may involve changes in PI3K/AKT/mTOR pathway activity (39–41). Although the autophagy process and PI3K/AKT/mTOR pathway seem to play a vital role in BCa progression, it is still unclear whether RAC3 is involved and what the underlying mechanisms are. Our study aims to fill the gap in the mechanism of RAC3 action on BCa metastasis and progression, which may lay the foundation for developing novel therapeutic strategies for BCa.

In this study, we comprehensively investigated the expression of RAC3 in multiple independent cohorts of BCa patients and determined the correlation of RAC3 expression with clinicopathological features and its prognostic value. We then used BCa cell lines and animal models to systematically investigate the functional roles of RAC3 in BCa cell proliferation, migration, and autophagy. We found that RAC3 knockdown in BCa cells increased autophagy flux *via* the PI3K/AKT/mTOR pathway. Manipulation of autophagy with multiple compounds shows that RAC3 interferes with the biological behavior of BCa cells by affecting autophagy flux. In conclusion, this study reports a novel role of RAC3 in mediating autophagy *via* the PI3K/AKT/mTOR pathway, providing new insights into the potential mechanism by which RAC3 promotes BCa genesis and metastasis.

## Material and Methods

### Bioinformatics Analysis

TCGA (42), GTEx (43), GEO (44) (GSE13507, GSE37815, GSE121711, GSE32849), and CCLE (45) databases were the sources of the transcriptome data of tumor samples, non-tumor samples, and cell lines. Differences between groups and survival analysis were performed. The patient characteristics of Bladder Urothelial Carcinoma (BLCA) from TCGA were summarized in **Table 1**.

**Table 1:** Relationship between different expression groups of RAC3 and clinical characteristics in bladder cancer in TCGA.

Characteristic	Low expression of RAC3	High expression of RAC3	P	statistic	method
n	204	204			
**Age, meidan (IQR)**	69 (60, 76)	68 (60, 76)	0.692	/	Wilcoxon
**Gender, n (%)**			0.822	0.05	Chisq.test
Female	55 (13.5%)	52 (12.7%)			
Male	149 (36.5%)	152 (37.3%)			
**Smoker, n (%)**			< 0.001	11.19	Chisq.test
No	70 (17.7%)	39 (9.9%)			
Yes	128 (32.4%)	158 (40%)			
**Histologic grade, n (%)**			0.007	7.29	Chisq.test
High Grade	185 (45.7%)	199 (49.1%)			
Low Grade	17 (4.2%)	4 (1%)			
**Pathologic stage, n (%)**			0.049	/	Fisher.test
Stage I	1 (0.2%)	1 (0.2%)			
Stage II	77 (19%)	53 (13.1%)			
Stage III	66 (16.3%)	74 (18.2%)			
Stage IV	59 (14.5%)	75 (18.5%)			
**OS event, n (%)**			0.003	8.96	Chisq.test
Alive	130 (31.9%)	99 (24.3%)			
Dead	74 (18.1%)	105 (25.7%)			
**DSS event, n (%)**			0.008	6.98	Chisq.test
Alive	150 (38.1%)	122 (31%)			
Dead	49 (12.4%)	73 (18.5%)			
**PFI event, n (%)**			0.004	8.43	Chisq.test
Alive	132 (32.4%)	102 (25%)			
Dead	72 (17.6%)	102 (25%)			

### Cell Culture and Reagents

The cell lines used in the study were listed in **Supplementary Table S1**. Lentiviruses for RAC3 knockdown and overexpression were purchased separately from Tsingke Co. Ltd. (Chongqing, China) and GenePharma Co. Ltd. (Shanghai, China). Oligonucleotides used for cloning were listed in **Supplementary Table S2**. Rapamycin (S1039), 3-Methyladenine (3-MA, S2767A) Bafilomycin A1 (Baf-A1, S1413) were purchased from Selleck Chemicals (Shanghai, China). MHY1485 (B5853) was purchased from APExBIO Technology (Houston, TX, USA).

### Immunohistochemistry (IHC) and Immunofluorescence(IF)

IHC and IF were performed as previously described (46). The protein expression was detected and scored as a Histochemistry score (H-Score). H-Score=∑(pi×i)=(percentage of weak intensity×1)+(percentage of moderate intensity×2)+(percentage of strong intensity×3). pi represents the percentage of positive signal pixel area/cell number; i stands for dye intensity).

Red-conjugated phalloidin (Actin-Tracker Red, Beyotime, China) and mCherry-GFP-LC3B plasmid were used in IF assay to detect F-actin and LC3 puncta. Images were acquired with a Zeiss fluorescence microscope (Axio Imager M2, Germany).

### RNA-Sequencing Study and Enrichment Analysis

Sequencing analysis (https://www.ncbi.nlm.nih.gov/bioproject/PRJNA824682) was performed on three biological replicates of 5637-shNC and 5637-shRAC3 cells (Tsingke Co. Ltd. Chongqing, China). Online website Metascape (47) was used to execute functional enrichment on differentially expressed genes (DEGs), including Kyoto Encyclopedia of Genes and Genomes (KEGG), HALLMARK, and Gene Ontology (GO, including Biological Process, Molecular Function, and Cellular Component groups) sets. Only terms with P<0.01, a minimum count of 3, and an enrichment factor >1.5 were considered significant.

### Cell Proliferation and Migration Assay

CCK-8 and colony formation assay were used for the proliferation test. Wound-healing and transwell migration assay were used for the migration test (48).

### Cell Adhesion

Cells in matrigel (BD Biosciences, California, USA)-coated plates were monitored by Lionheart FX Automated Live Cell Imager (Agilent Technologies, Inc. USA). Cells were digested into single cells and replaced in Matrigel-coated plates. The cells gradually adhere to the plate wall and spread, and can be distinguished from non-adherent (spherical or ellipsoidal) cells. Time-lapse videos were captured and shown in **Supplementary Video 1**.

### Tumourigenicity Assays and *In Vivo* Metastasis Assay

Tumourigenicity assays: 1x10^6^ of 5637-shNC/shRAC3 cells (50μl) were injected into the forelimbs of four-week-old nude mice. The tumor volumes were assessed every 5–8 days as follows: Volume (mm^3^) = (length × width^2^)/2 (49). 53 days after, all mice were sacrificed by cervical dislocation injection. The tumors were dissected, followed by IHC staining.

*In vivo* tumor metastasis assay: 2x10^6^ of 5637-shNC or 5637-shRAC3 cells (100μl) were tail-vein injected into the four-week-old NOD-SCID mice. 55 days later, all mice were sacrificed. The lungs were dissected for H&E staining. All animal studies were performed according to the guidelines approved by the Laboratory Animal Welfare and Ethics Committee of the Third Military Medical University (Chongqing, China).

### Western Blot Analysis, Flow Cytometry, and Transmission Electronic Microscopy (TEM)

We performed WB assay (48), flow cytometry (50), and TEM assay (51) as previously described. The antibodies were listed in **Supplementary Table S3**. Flow cytometry was used to analyze the cell cycle and apoptosis. Images of TEM assay were acquired using an electron microscope (TECNAI 10, Philips, Netherlands).

### Statistical Analyses

Statistical results were calculated using R software (version 3.6.3). The data shown are representative of three independent experiments and expressed as mean ± SD. Student’s t-test or Wilcoxon rank-sum test by ggplot2 package was used to analyze intergroup differences. The survival probability was evaluated with the Kaplan–Meier method (log-rank test) by survminer and survival packages. ROC curve was plotted, and the area under the curve (AUC) was calculated using the pROC package. Groups for clinical-pathological variables were analyzed with chi-squared tests. P < 0.05 was considered statistically significant.

## Results

### Analysis of GTEx+TCGA+GEO Datasets Indicates That RAC3 Is Highly Expressed and Predicts a Poor Prognosis of BCa Patients

**Figure 1:**
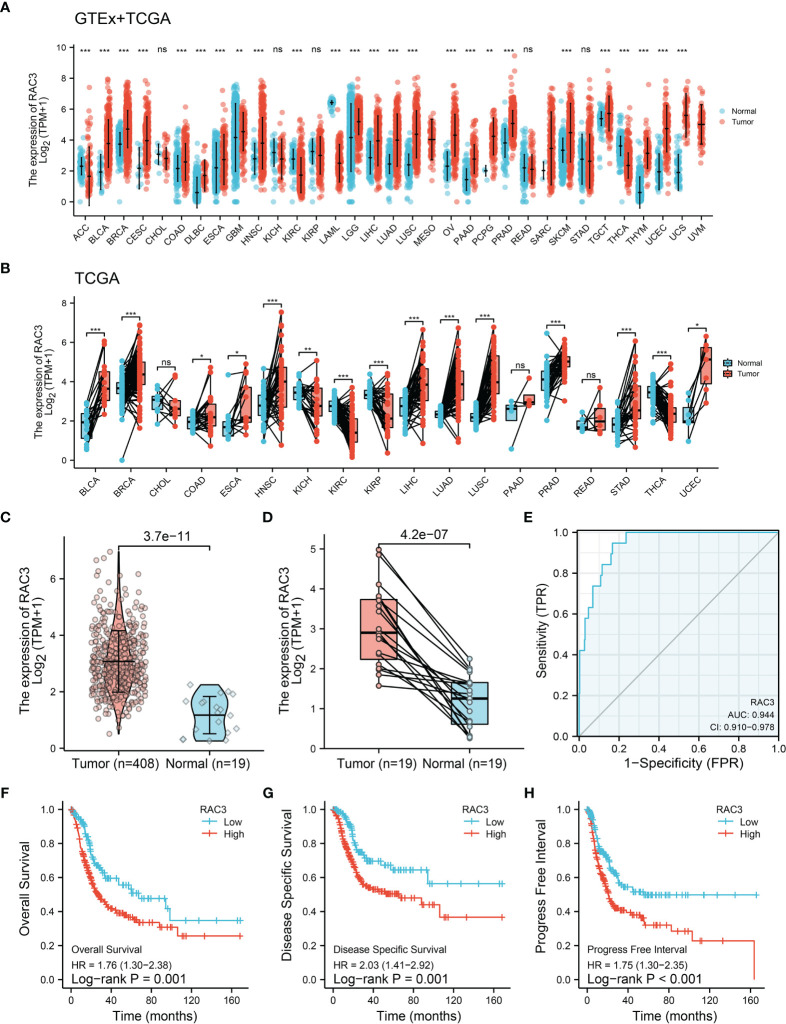
TCGA+GETx analysis: RAC3 is highly expressed and predicts poor prognosis of BCa patients. **(A)** mRNA expression of RAC3 in various cancers from the TCGA and GETx database (unpaired samples). **(B)** mRNA expression of RAC3 in various cancers from the TCGA database (paired samples). **(C)** mRNA expression of RAC3 in BLCA (unpaired samples). **(D)** mRNA expression of RAC3 in BLCA (paired samples). **(E)**. Diagnostic efficacy testing by AUC-ROC analysis in BLCA. **(F)** Overall Survival curve of tumor and normal groups in BLCA. **(G)** Disease Specific Survival curve of tumor and normal groups in BLCA. **(H)** Progress Free Interval curve of tumor and normal groups in BLCA. Bars/Boxes represent the median, 25th and 75th percentiles. ns, not significant. *P < 0.05, **P < 0.01, ***P < 0.001. ACC, Adrenocortical carcinoma; BLCA, Bladder Urothelial Carcinoma; BRCA, Breast invasive carcinoma; CESC, Cervical squamous cell carcinoma and endocervical adenocarcinoma; CHOL, Cholangiocarcinoma; COAD, Colon adenocarcinoma; DLBC, Lymphoid Neoplasm Diffuse Large B-cell Lymphoma; ESCA, Esophageal carcinoma; GBM, Glioblastoma multiforme; HNSC, Head and Neck squamous cell carcinoma; KICH, Kidney Chromophobe; KIRC, Kidney renal clear cell carcinoma; KIRP, Kidney renal papillary cell carcinoma; LAML, Acute Myeloid Leukemia; LGG, Brain Lower Grade Glioma; LIHC, Liver hepatocellular carcinoma; LUAD, Lung adenocarcinoma; LUSC, Lung squamous cell carcinoma; MESO, Mesothelioma; OV, Ovarian serous cystadenocarcinoma; PAAD, Pancreatic adenocarcinoma; PCPG, Pheochromocytoma and Paraganglioma; PRAD, Prostate adenocarcinoma; READ, Rectum adenocarcinoma; SARC, Sarcoma; SKCM, Skin Cutaneous Melanoma; STAD, Stomach adenocarcinoma; TGCT, Testicular Germ Cell Tumors; THCA, Thyroid carcinoma; THYM, Thymoma; UCEC, Uterine Corpus Endometrial Carcinoma; UCS, Uterine Carcinosarcoma; UVM, Uveal Melanoma.

Sequencing data analysis of GTEx+TCGA showed the RAC3 mRNA was higher in most tumors than that in non-tumor tissues, whether in all samples (**Figure 1A**) or paired samples (**Figure 1B**). RAC3 expression in BCa tissues was higher than that in normal bladder tissues (**Figure 1C**, P=3.7-e-11).

**Figure 2:**
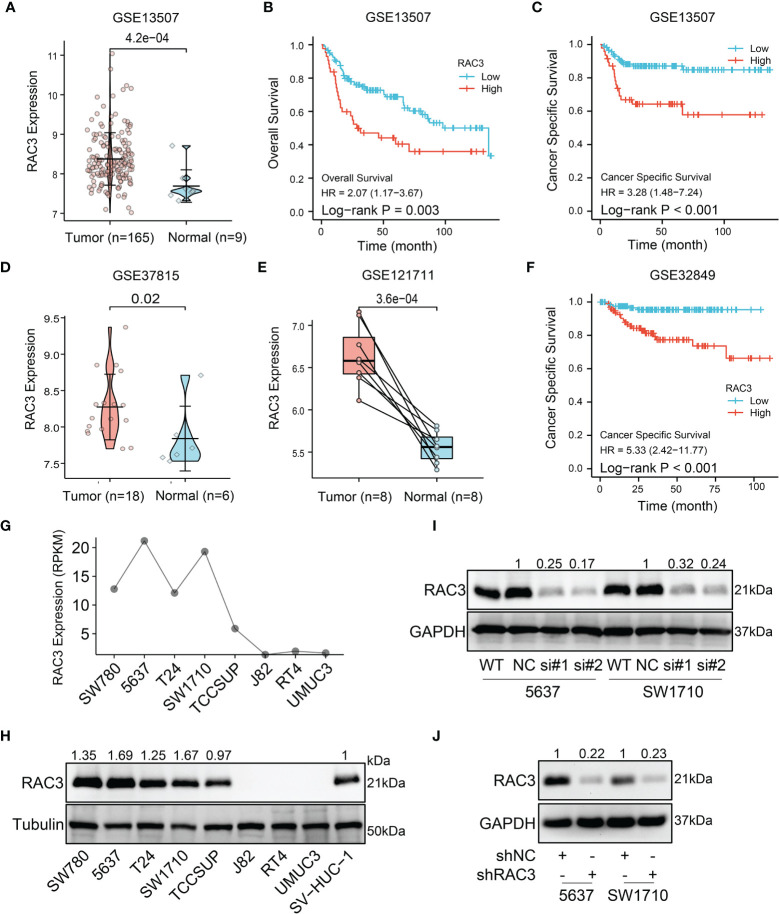
GEO and cell lines analysis: RAC3 is highly expressed in BCa samples and cells and predicts poor prognosis **(A)** mRNA expression of RAC3 in GSE13507 (unpaired samples) **(B)** Overall Survival curve of tumor and normal groups in GSE13507. **(C)** Cancer Specific Survival curve of tumor and normal groups in GSE13507. **(D)** mRNA expression of RAC3 in GSE37815 (unpaired samples) **(E)** mRNA expression of RAC3 in GSE121711 (paired samples). **(F)** Cancer Specific Survival curve of tumor and normal groups in GSE32849 **(G)** mRNA expression of RAC3 in CCLE database **(H)** Western blotting analysis of RAC3 basal protein expression in 8 BCa cell lines and 1 ureteral epithelium cell (SV-HUC-1); Tubulin was used as a loading control **(I)** Western blotting analysis of RAC3 protein expression in 5637 and SW1710 cells transfected with siNC and siRAC3 #1, #2. GAPDH was used as a loading control **(J)** Western blotting analysis of RAC3 protein expression in 5637 and SW1710 cells infected with shNC and shRAC3 lentivirus. GAPDH was used as a loading control.

We also analyzed the expression of RAC3 in subgroups from different clinical characteristics (**Figures S1A-F, J**) and found that RAC3 expression progressively increases with cancer progression. The more serious the disease, the higher RAC3 expressed. Considering individual differences, the analysis of 19 paired samples also showed that RAC3 expression in BCa tissues was higher (**Figure 1D**, P=4.2e-07). The mRNA level of RAC3 had good diagnostic accuracy, with an AUC of ROC reached 0.944 (**Figure 1E**, 95% CI = 0.910-0.978), which could better discriminate tumor and non-tumor samples. Survival analysis showed that Overall Survival (**Figure 1F**, P=0.001), Disease Specific Survival (**Figure 1G**, P=0.001), and Progress Free Interval (**Figure 1H**, P<0.001) were significantly shorter in patients with high RAC3 expression than in those with low expression. Chi-square tests confirmed differences in terms of Smoking history, Pathologic stage, Histologic grade, OS event, DSS event, PFI event (P<0.05, **Table 1**). A comparison of RAC3 expression between different prognoses illustrated the worse survival outcome accompanied by higher RAC3 expression (**Figures S1G-I**). In other words, highly expressed RAC3 in BCa is associated with heavier smoking history, higher grade, higher stage, and worse prognosis. We searched multiple GEO datasets assisted in the validation of the above results. Differential analysis in **Figure 2A** (GSE13507, P=4.2e-04), **Figure 2D** (GSE37815, P=0.02), **Figure 2E** (GSE121711, P=3.6e-04) validated that RAC3 was highly expressed in BCa. Survival analysis of Overall Survival (**Figure 2B**, P=0.003) and Cancer Specific Survival (**Figure 2C**, P<0.001) from GSE13507, as well as Cancer Specific Survival (**Figure 2F**, P<0.001) provided by GSE32849 validated the poor outcome of patients with high RAC3 expression.

### Knockdown RAC3 Inhibits the Proliferation of BCa Cells *In Vitro* and *In Vivo*

**Figure 3:**
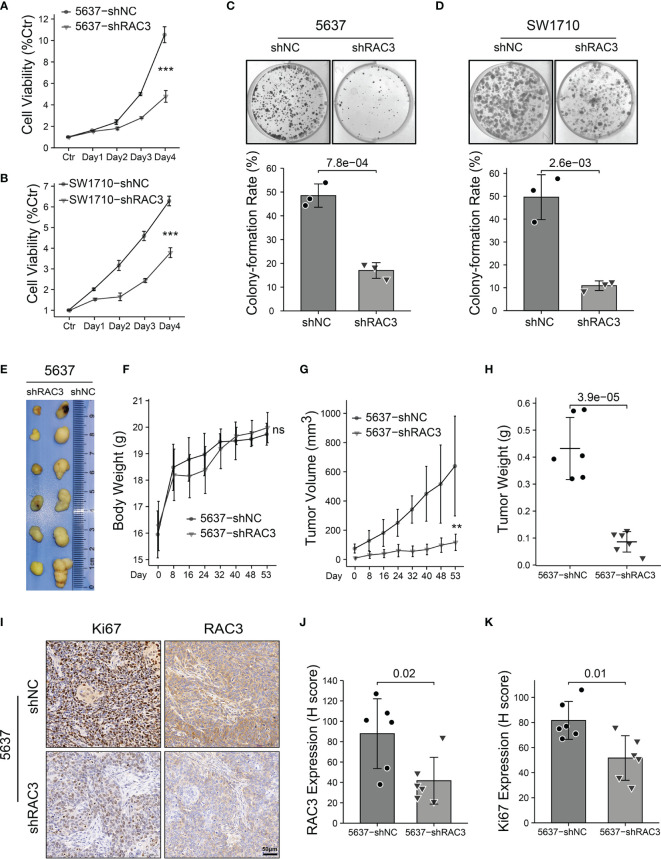
Knockdown RAC3 inhibits proliferation of BCa cells *in vitro* and *in vivo*. **(A, B)** CCK8 assay comparing the proliferation of 5637 **(A)** and SW1710 **(B)** cells. 10^3^ cells were seeded per well in 96-well plates. Absorbance at 450 nm was detected with 10% CCK-8, which incubated for 2 hours each day for five days. Cell viability was assessed as a percentage of Ctr (Day0). Significance is indicated versus shNC groups. **(C, D)** Colony formation assay comparing the proliferation of 5637 **(C)** and SW1710 **(D)** cells. 10^3^ cells per well were seeded in 6-well plates and cultured for ten days. Plates were washed and fixed with 4% paraformaldehyde, stained with crystal violet for 30 min. colony-formation rate (%)= (number of the cells seeded−number of the colons counted at day 10)/number of the cells seeded × 100%. **(E-H)** Images of the xenograft tumors from 5637-shNC or 5637-shRAC3 cells subcutaneously injected nude mice **(E).** Nude mice weight curves **(F)** and tumor growth curves **(G)** were plotted. Tumor weights were compared between 5637-shNC and 5637-shRAC3 groups **(H)**. **(I-K)** Representative images of IHC staining of RAC3 and Ki67 in tumor xenografts **(I)**, protein expressions of RAC3 **(J)** and Ki67 **(K)** were compared in 5637-shNC and 5637-shRAC3 groups based on the H-score. ns, not significant. **P < 0.01, ***P < 0.001.

CCLE database analysis (**Figure 2G**) and WB test (**Figure 2H**) detected RAC3 expression in multiple BCa cell lines and found that the mRNA level was consistent with protein level. 5637 and SW1710 cells with higher RAC3 expression were picked out to be transfected with two siRNAs/shRNAs, specifically targeting RAC3 to inhibit its expression (**Figures 2I** and **S2A**), and the maximum knockdown efficiency was observed with sh#2 (hereafter referred to as shRAC3) and was used for all subsequent studies (**Figure 2J**). CCK8 assay indicated that knockdown RAC3 could inhibit the proliferation of 5637 and SW1710 cells (**Figures 3A, B** and **S2B**). The Colony formation assay results further confirmed the inhibitory effect (**Figures 3C, D**). A small amount of 5637 cell lysate was used as a positive control to detect the three cell lines with low expression of RAC3 (**Figure S3A**). J82 infected with overexpressing RAC3 lentivirus (**Figure S3B**) produced a proliferation-promoting effect (**Figure S3CD**). Overexpress RAC3 in shRAC3 cells confirmed that the defects of cell proliferation in shRAC3 cells is not due to the off-target effects (**Figure S2AB**).

Based on the *in vitro* results, the *in vivo* proliferation suppressive effect of RAC3 depletion was determined in a xenograft tumor model by subcutaneous injection of 5637-shNC/shRAC3 cells (**Figure 3E**). Without affecting the body weight of the node-mice (**Figure 3F**), the tumor volume (**Figure 3G**) and weight (**Figure 3H**) were significantly reduced in the 5637-shRAC3 group. In addition, IHC staining showed that compared with 5637-shNC, the 5637-shRAC3 group had a lower Ki67 protein level (**Figures 3I-K**). In summary, knockdown RAC3 inhibited the proliferation of BCa cells.

### Knockdown RAC3 Attenuated the Migration and Adhesion of BCa Cells

**Figure 4:**
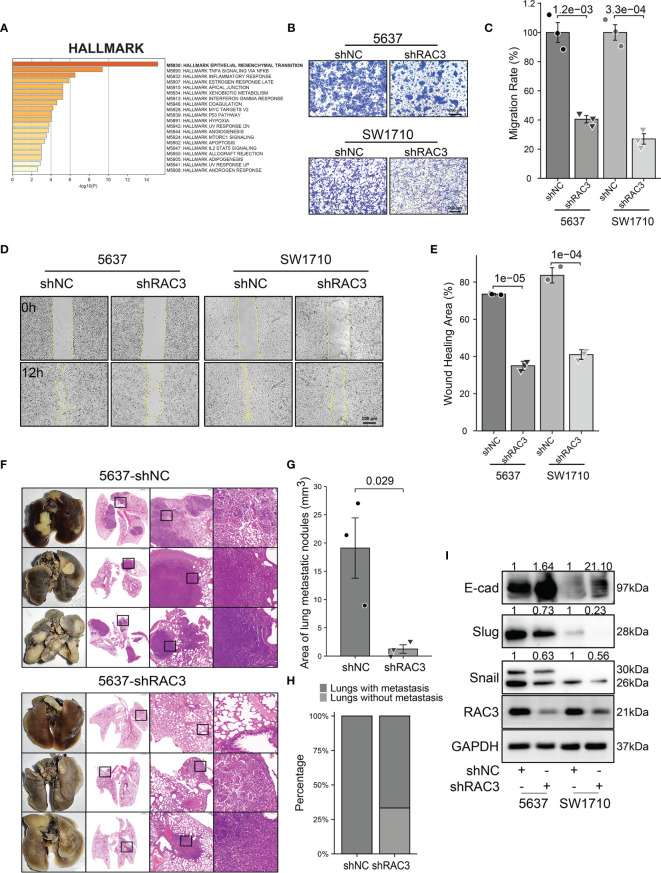
Knockdown RAC3 attenuated the migration of BCa cells. **(A)** HALLMARK analysis showing DEGs between 5637-shNC and 5637-shRAC3 cells were enriched in EPITHELIAL MESENCHYMAL TRANSITION process. **(B, C)** Comparison of the migration of 5637 and SW1710 cells using transwell compartments. 1x10^5^ 5637 cells and 0.5x10^5^ SW1710 cells were resuspended in 200 μl serum-free medium using the transwell compartments (Corning, USA), 800 μl complete medium was added in the lower chamber. The cells on the chamber’s upper surface were washed, while those on the lower surfaces were fixed with 4% paraformaldehyde and stained with crystal violet after 12h for SW1710 cells and 36h for 5637 cells. **(D, E)** Comparison of the migration 5637 and SW1710 cells using Wound-healing assay. cells were seeded into the Culture-Inserts 2 Well system (Ibidi GmbH, Germany). The images were acquired at 0h and 12h after the removal of the inserts. Wound-healing area (%) = (area of the scratch at 0h−area of the scratch at 12h)/area of the scratch at 0h×100%. **(F-H)** Lung tissues and HE staining Images from 5637-shNC or 5637-shRAC3 cells tail-vein injected NOD-SCID mice **(F)** Area of lung metastatic nodules were compared between 5637-shNC and 5637-shRAC3 groups **(G)**. Lung metastasis rate from two groups **(H)(I)** Western blotting analysis of the E-cadherin, Slug, Snail protein expression in 5637-shNC/shRAC3 and SW1710-shNC/shRAC3 cells. GAPDH was used as a loading control. Quantification of protein expression is shown in **Figure S10A**.

The sequencing results of 5637-shNC/shRAC3 showed 692 DEGs among two groups, which were enriched into the EMT process in the HALLMARK dataset (**Figure 4A**). Transwell migration assay found that compared with 5637-shNC/SW1710-shNC, the number of 5637-shRAC3/SW1710-shRAC3 cells passing through the chamber was significantly reduced (**Figures 4B, C**). The wound-healing assay revealed that 5637-shRAC3/SW1710-shRAC3 cells migrated significantly slower (**Figures 4D, E**). Further *in vivo* experiments showed that the lung metastasis rate and the area of metastatic nodules in the 5637-shRAC3 group were significantly reduced by tail-vein injection of 5637-shNC/shRAC3 cells into NOD-SCID mice, which further confirmed the anti-metastatic effect *in vitro* (**Figures 4F–H**). WB analysis of hallmark molecules of EMT also demonstrated that snail and slug proteins decreased, and a concomitant E-cadherin protein increased in 5637-shRAC3/SW1710-shRAC3 cells (**Figures 4I** and **S10A**). To sum up, these data suggest that RAC3 attenuation inhibits the migratory ability of BCa cells. Correspondingly, promotion of migration was observed in J82-RAC3 cells (**Figure S3E**). Overexpress RAC3 in shRAC3 cells confirmed that the defects of cell migration in shRAC3 cells is not due to the off-target effects (**Figures S2A, B**).

In addition, GO analysis of DEGs from sequencing data exhibited pronounced enrichment of regulation of cell adhesion, extracellular matrix, collagen binding, cell adhesion molecule binding, which were closely related to metastasis (**Figure S4A**). With the Automated Live Cell Imager, we did observe that the adhesion of 5637-shRAC3/SW1710-shRAC3 cells was weaker (Video 1). IF staining of F-actin revealed a cytoskeleton rearrangement of 5637-shRAC3/SW1710-shRAC3 cells with a marked reduction in filopodia and lamellipodia (**Figure S4B**), which may be one of the mechanisms contributing to the weakened cell migration.

### RAC3 Is a Regulator of the PI3K/AKT/mTOR Pathway, and Knockdown RAC3 Promotes Autophagy in BCa Cells

**Figure 5:**
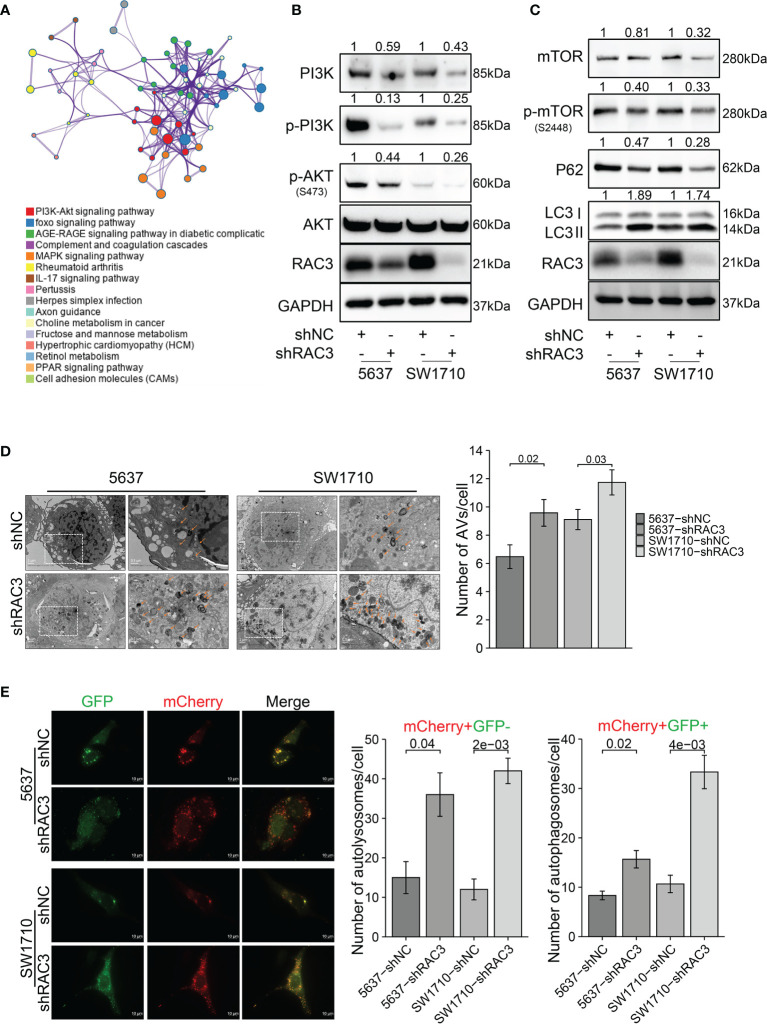
RAC3 is a regulator of the PI3K/AKT/mTOR pathway, and knockdown RAC3 promotes autophagy in BCa cells. **(A)** KEGG analysis showing DEGs between 5637-shNC and 5637-shRAC3 cells were enriched in PI3K/AKT pathway. **(B)** Western blotting analysis of the PI3K, p-PI3K, p-AKT, and AKT protein expression in 5637-shNC/shRAC3 and SW1710-shNC/shRAC3 cells. GAPDH was used as a loading control. Quantification of protein expression is shown in **Figure S10B**. **(C)** Western blotting analysis of the p-mTOR, mTOR, P62, and LC3 protein expression in 5637-shNC/shRAC3 and SW1710-shNC/shRAC3 cells. GAPDH was used as a loading control. Quantification of protein expression is shown in **Figure S10B**. **(D)** Representative electron micrographs of autophagic vesicles in 5637-shNC/shRAC3 and SW1710-shNC/shRAC3 cells. The number of autophagic vacuoles (AVs), including autophagosomes and autolysosomes, in each cell was quantified. **(E)** The mCherry-GFP-LC3B fusion assay. Immunofluorescence images of 5637-shNC/shRAC3 and SW1710-shNC/shRAC3 cells transfected with mCherry-GFP-LC3B plasmids for 24h. The number of yellow LC3 puncta (representing autophagosomes) and red LC3 puncta (representing autolysosomes) were quantified.

Flow cytometry analysis indicated that knockdown RAC3 caused cycle arrest with a significant increase in the G2 phase cells (**Figure S5A**) and no significant difference in apoptosis rate (**Figure S5B**), suggesting that it is not apoptosis caused proliferation and migration inhibition in 5637-shRAC3/SW1710-shRAC3 cells. KEGG analysis of DEGs suggested significant alteration of the PI3K/AKT pathway (**Figure 5A**). The effect of RAC3 on the pathway was confirmed by reduced p-PI3K, p-AKT protein levels in 5637-shRAC3/SW1710-shRAC3 cells (**Figures 5B** and **S10B**). Analysis of TCGA-BLCA corroborated that AKT/mTOR is indeed positively correlated with RAC3 expression (**Figure S6**). Further WB experiments found that RAC3 knockdown downregulated mTOR, p-mTOR and, conversely, increased LC3II (**Figures 5C** and **S10B**). It is suggested that RAC3 is a regulator of the PI3K/AKT/mTOR pathway. TEM assay showed more AVs in the cytoplasm of 5637-shRAC3/SW1710-shRAC3 cells compared to the shNC groups (**Figure 5D**). The mCherry-GFP-LC3B fusion assay also found that the number of autophagosomes and autolysosomes were both higher in shRAC3 cells than shNC cells (**Figure 5E**). Moreover, we used Baf A1 to perform an autophagy flux assay and observed that LC3II protein expression levels were further increased by Baf A1 (**Figure S7**). These data suggest that RAC3 knockdown can increase the formation of autophagosomes upstream of the lysosomes. Collectively, our data demonstrated that knockdown RAC3 positively regulates autophagy. Nevertheless, inconsistent with expectation, overexpression of RAC3 positively affected J82 cells (**Figures S3B–E**), but no significant changes were found in PI3K/AKT/mTOR/P62/LC3 proteins (**Figure S3F**). Our data suggest that knockdown RAC3 promotes autophagy in BCa cells.

### Suppress Autophagy Protects BCa Cells From Proliferation and Migration Inhibition Induced by RAC3 Knockdown

**Figure 6:**
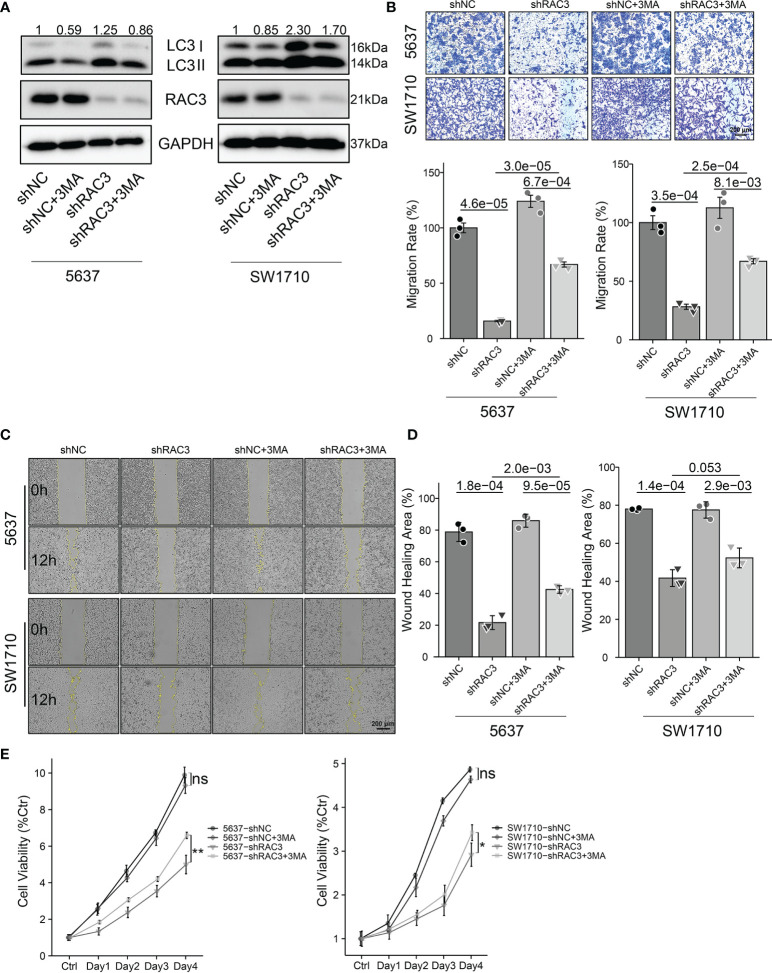
Inhibition of autophagy protects BCa cells from proliferation and migration inhibition induced by RAC3 knockdown. 5637-shNC/shRAC3 and SW1710-shNC/shRAC3 cells were in the presence or absence of 3MA (5mM) for 2h. **(A)** Western blotting analysis of P62 and LC3 protein expression. GAPDH was used as a loading control. Quantification of protein expression is shown in **Figure S10C**. **(B)** Comparison of the migration using transwell compartments as described in **Figure 4**. **(C, D)** Wound-healing assay comparing the motility of cells as described in **Figure 4**. **(E)**CCK8 assay comparing the cell viability as described in **Figure 3** ns, not significant. *P < 0.05, **P < 0.01.

We used 3MA, an inhibitor of autophagy, to evaluate whether the inhibition of proliferation and migration observed in 5637-shRAC3/SW1710-shRAC3 cells was caused by the upregulation of autophagy. WB showed that 3MA significantly decreased RAC3 inhibition-dependent LC3II protein expression (**Figures 6A** and **S10C**). Wound healing and transwell migration assays revealed that the metastasis suppressing effect resulting from RAC3 inhibition was reversed (**Figures 6B–D**). CCK8 assay revealed that 3MA could reverse the proliferation attenuation phenotype of 5637-shRAC3/SW1710-shRAC3 cells (**Figure 6E**). The above results indicated that autophagy inhibition reverses the anti-metastatic and anti-proliferative effects induced by RAC3 knockdown. Thus, our results suggest that knockdown RAC3 leads to migration and proliferation inhibition of BCa cells by promoting autophagy.

### RAC3 Regulates Autophagy Through mTOR

**Figure 7:**
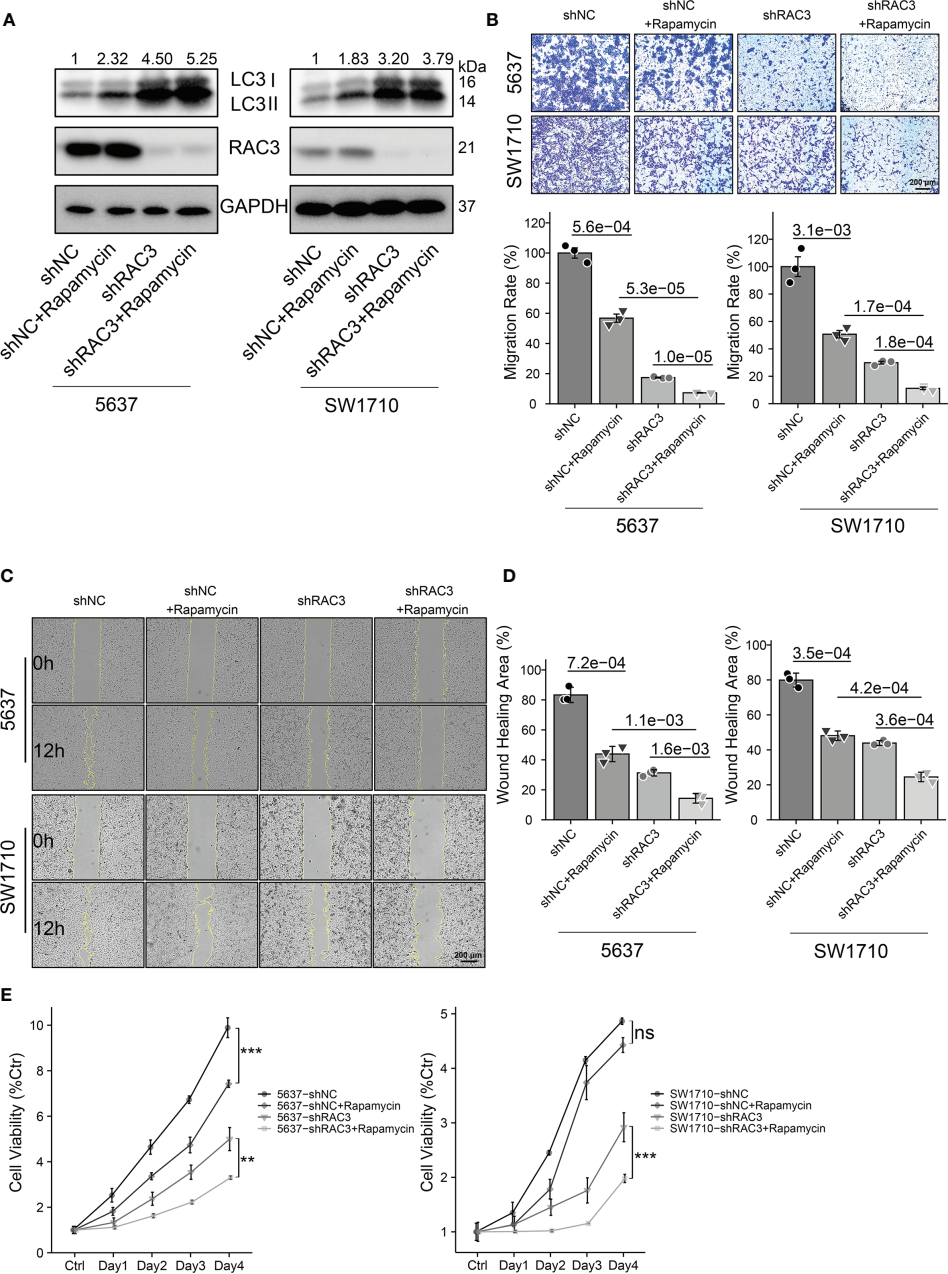
Dual inhibition of RAC3 and mTOR enhances autophagy and exerts synergistic anti-metastatic and anti-proliferative effects on BCa cells. 5637-shNC/shRAC3 and SW1710-shNC/shRAC3 cells were in the presence or absence of Rapamycin (100nM) for 24h. **(A)** Western blotting analysis of P62 and LC3 protein expression. GAPDH was used as a loading control. Quantification of protein expression is shown in **Figure S10D**. **(B)** Comparison of the migration using transwell compartments as described in **Figure 4**. **(C, D)**Wound-healing assay comparing the motility of cells as described in **Figure 4**. **(E)**CCK8 assay comparing the cell viability as described in **Figure 3**. ns, not significant. **P < 0.01, ***P < 0.001.

Considering the relationship between RAC3, mTOR, and autophagy, we then investigated whether targeting RAC3 and mTOR in combination would further impair BCa cells’ malignancy. We observed that Rapamycin, an mTOR inhibitor, further increased LC3II protein expression based on RAC3 knockdown compared with 5637-shNC/SW1710-shNC cells (**Figures 7A** and **S10D**). Consistent with the effect of RAC3 knockdown, Rapamycin alone resulted in decreased cell migration. Rapamycin could further enhance the inhibitory effect of RAC3 knockdown (**Figures 7B–D**). CCK8 assay found that Rapamycin alone inhibited the proliferation of 5637. The mTOR inhibition further attenuated the proliferation of 5637-shRAC3/SW1710-shRAC3 cells, resulting in a synergistic anti-proliferative effect (**Figure 7E**). The above results demonstrated that combined inhibition of RAC3 and mTOR significantly increased autophagy activity, resulting in synergistic anti-metastatic and anti-proliferative effects in 5637 and SW1710 cells. Moreover, we used MHY1485, a TOR activator, to evaluate whether mTOR activation could rescue the autophagy and migration phenotype in RAC3-knockdown cells (**Figure S8**). WB showed that MHY1485 significantly decreased RAC3 inhibition-dependent LC3II protein expression (**Figure S8A**). Transwell assay revealed that the migration suppressing effect was partially rescued by MHY1485 (**Figure S8B**). The above results indicated that mTOR activation results in autophagy inhibition and reverses the anti-migration effects induced by RAC3 knockdown. To verify the direct effect of RAC3 on mTOR, we used immunofluorescence to determine whether RAC3 and mTOR colocalize (**Figure S9**). The colocalization was dispersed in the cytoplasm and slightly aggregated around the perinuclear region (but not at the plasma membrane), which would support a possible direct regulation of mTOR by RAC3. Upon RAC3 knockdown, the anti-RAC3 staining weakened as expected, but no significant changes were observed in mTOR localization. Combined, we suggest that the RAC3 inhibition induces autophagy to impair the migration of bladder cancer cells *via* the PI3K/AKT/mTOR pathway.

## Discussion

This study demonstrated the effects and possible mechanisms of RAC3 in BCa progression. Multiple datasets and cell experiments confirmed that RAC3 was highly expressed in BCa tissues and cells. Moreover, the high expression of RAC3 was associated with higher grading/staging and poor prognosis, indicating a potential correlation with the carcinogenesis of BCa. Further *in vitro* and *in vivo* models confirm that knockdown RAC3 inhibits autophagy-mediated proliferation and migration of BCa cells through the PI3K/AKT/mTOR pathway. Rearrangement of the cytoskeleton, reduction of lamellipodia and filopodia, and cell cycle arrest were observed in 5637-shRAC3/SW1710-shRAC3 cells, which have not previously been reported. All these results indicate that RAC3 is a valuable biomarker of BCa.

Studies have suggested that knockdown RAC3 promotes apoptosis in lung cancer cells (52, 53). However, no difference in apoptosis was observed when proliferation and migration were impaired in RAC3 knockdown BCa cells, which prompted us to consider whether knockdown RAC3 induced non apoptosis-dependent cell death. DEGs between 5637-shNC and 5637-shRAC3 groups were used to conduct the KEGG analysis and showed an enrichment of the PI3K/AKT/mTOR pathway, suggesting that RAC3 may affect BCa progression by regulating autophagy. In most cases, autophagy is a survival mechanism, and uncontrolled activation of autophagy can lead to cell disassembly with death (54, 55). Furthermore, this form of death can be apoptosis and necroptosis independent (56). Despite discovering different individuals involved in the autophagy process and regulation, the whole picture of autophagy regulation remains a mystery. Identifying RAC3 as an autophagy regulator has opened up new avenues for autophagy research. For example, autophagy was induced by RAC3 knockdown in HCT116, HeLa, MDA-MB-231, PC3, U87 (57), and HUVEC (58) cells, but whether RAC3 was involved in the tumourigenesis of BCa and the underlying mechanism was not clear.

In our study, knockdown RAC3 impaired AKT activation, but the AKT activator SC-79 did not rescue the anti-proliferative/anti-migratory effect of RAC3 knockdown cells (data not shown), which may be explained by the inability of SC79 to activate AKT when PI3K is inhibited (59). Decreased p-mTOR and increased LC3II protein levels, and increased numbers of AVs and LC3 puncta, characteristic of autophagy pathway activation, were detected in 5637-shRAC3/SW1710-shRAC3 cells. The effect of RAC3 inhibition on BCa cells’ proliferation and migration was partially reversed by the autophagy inhibitor 3MA, confirming the role of autophagy in these processes. Several studies have suggested that the incapacitation of autophagy is essential for tumorigenesis (34). Autophagy is a tumor-suppressive mechanism that maintains genomic integrity and suppresses tumorigenesis (60, 61), as occurs with RAC3 knockdown cells. Activation of PI3K/AKT/mTOR, an important pathway regulating autophagy, is prevalent in many cancers and may be responsible for reducing cytoprotective autophagy and deregulated proliferation (62). Studies have confirmed that targeting autophagy in combination with other treatment strategies is generally more effective than monotherapy (63). Experiments demonstrated that dual inhibition of RAC3 and mTOR enhanced autophagy and exerted synergistic anti-metastatic and anti-proliferative effects on BCa cells compared to either mode of inhibition alone. Therefore, this combined targeting is a potential strategy for BCa treatment. Furthermore, mTOR agonists reduced the LC3II protein level and rescued the anti-metastatic effects on shRAC3 cells. mTOR and RAC3 were colocalized aggregated around the perinuclear region (but not at the plasma membrane), which would support a possible direct regulation of mTOR by RAC3. Upon RAC3 knockdown, the anti-RAC3 staining weakened as expected, but not like the effect of RAC1 on mTOR (64), no significant changes were observed in mTOR localization. Combining all the results, we suggest that RAC3 functions are associated with the regulation of mTOR activation and autophagy formation. Our finding that autophagy manipulation affects the migration of shRAC3 cells updates the study by Wan Long Zhu et al. (57), who found that RAC3 expression levels negatively correlate with basal autophagy levels. However, it is also important to note that Rapamycin inhibited the proliferation of 5637-shNC cells and 3MA inhibited the proliferation of 5637-shNC/SW1710-shNC cells, again confirming that sustainable cell growth can be achieved only with appropriate autophagy intensity (65). Also, let us realize that autophagy regulation in BCa cells is highly context-specific. This dependency can be understood as cancer cells being sensitive to autophagy induction at a basal level and more sensitive to inhibition at higher autophagy levels. The effects of autophagy differ in different cancer types and different cell lines. Various opposing explanations challenge the effect of autophagy on the proliferation of tumor cells. Such as inhibition of autophagy leading to regression of pancreatic cancer (66) and lung cancer (67) suggests a role for autophagy in sustaining tumor growth. While the induction of autophagy in renal cell cancer (68), colorectal cancer (69) inhibits cell proliferation, suggesting that excessive autophagy can limit cell growth. Our data and others suggest that significant deviations from the physiological set point of basal autophagy, either high or low, can lead to cellular toxicity. This result enables us to understand the necessity of identifying autophagy regulatory factors in pursuing tumor-targeted therapy. RAC signalling may also have many crosstalk and connections with the Wnt pathway, the Ras pathway, etc. Extensive studies are needed to delineate the complete RAC3 signalling in autophagy regulation. Identifying RAC3 has vital implications for understanding RAC isoform-specific functions and identifying therapeutic targets.

EMT is critical for tumor metastasis, which is a process of reprogramming from an epithelial phenotype to an invasive mesenchymal-like phenotype. There is evidence that autophagy impairs EMT by promoting snail degradation (70). In our study, the EMT enrichment and E-cad/snail/slug protein changes suggest that the autophagy induced by RAC3 reduction contributes to the EMT impairment of BCa cells. However, the mechanism still needs to be deeply investigated. The experiment revealed pseudopodia and skeletal alterations in 5637-shRAC3/SW1710-shRAC3 cells, accompanied by poor cell adhesion, which was previously corroborated in breast cancer (18, 19). Focal adhesion signalling at the leading edge of pseudopodia is the initiating step in migration (71). Autophagy degradation of focal adhesion proteins leads to weaker migration (72). Taken together, the enhanced autophagy by knockdown of RAC3 may affect the BCa cells migration through multiple factors mentioned above. Collectively, our findings uncover a more detailed mechanism by which RAC3 promotes BCa progression.

However, this study also has certain limitations. First, there are already some studies showing that the active RAC3 form affects oncogenesis (21, 73–75), and it is reasonable to speculate that the downstream signal transmission will be affected if the activation state of RAC3 is altered in BCa cells, but it still needs proper validation. Second, additional studies are needed to confirm when does RAC3 begin to promote and to what extent it promotes bladder cancer occurrence (or progression). The selective expression and differentiated function of RAC3 in different biological backgrounds have important guiding significance for when to intervene, how to intervene, and how much to intervene on BCa patients. Further studies are required to make up for the limitations and fully elucidate these concerns.

## Conclusion

RAC3 is overexpressed in BCa tissues and cells. High expression of RAC3 is associated with advanced tumor characteristics and poor prognosis. We identified a novel mechanism underlying BCa cells’ proliferation and migration: the RAC3 negatively regulated PI3K/AKT/mTOR mediated autophagy. Combined targeting of RAC3 and mTOR provides a new option for the therapy of BCa patients.

## Data Availability Statement

The datasets presented in this study can be found in online repositories. The names of the repository/repositories and accession number(s) can be found in the article/**Supplementary Material**.

## Ethics Statement

The studies involving human participants were reviewed and approved by TCGA, GTEx, GEO (GSE13507, GSE37815, GSE121711, GSE32849), and CCLE databases. Written informed consent for participation was not required for this study in accordance with the national legislation and the institutional requirements. The animal study was reviewed and approved by Laboratory Animal Welfare and Ethics Committee of the Third Military Medical University.

## Author Contributions

ZC and JY designed the study; LW, SL, YH, and HD collected and assembled data. LW and JS performed the data analysis; BZ, YL, and WW contributed the analysis tools; and LW wrote the paper. All authors read and approved the final manuscript.

## Funding

This study was supported by the National Natural Science Foundation of China (Grants 81772738, 81572772, 81602250) and Key Talents Support Plan of Army Medical University (2019, 410301053410).

## Conflict of Interest

The authors declare that the research was conducted in the absence of any commercial or financial relationships that could be construed as a potential conflict of interest.

## Publisher’s Note

All claims expressed in this article are solely those of the authors and do not necessarily represent those of their affiliated organizations, or those of the publisher, the editors and the reviewers. Any product that may be evaluated in this article, or claim that may be made by its manufacturer, is not guaranteed or endorsed by the publisher.
