# Supplementary material for: RAC3 Inhibition Induces Autophagy to Impair Metastasis in Bladder Cancer Cells via the PI3K/AKT/mTOR Pathway
Source: Front Oncol. 2022 Jun 30;12:915240. doi: 10.3389/fonc.2022.915240 (PMC9279623; doi:10.3389/fonc.2022.915240)
Supplement: Supplementary file 1 [file DataSheet_1.pdf]

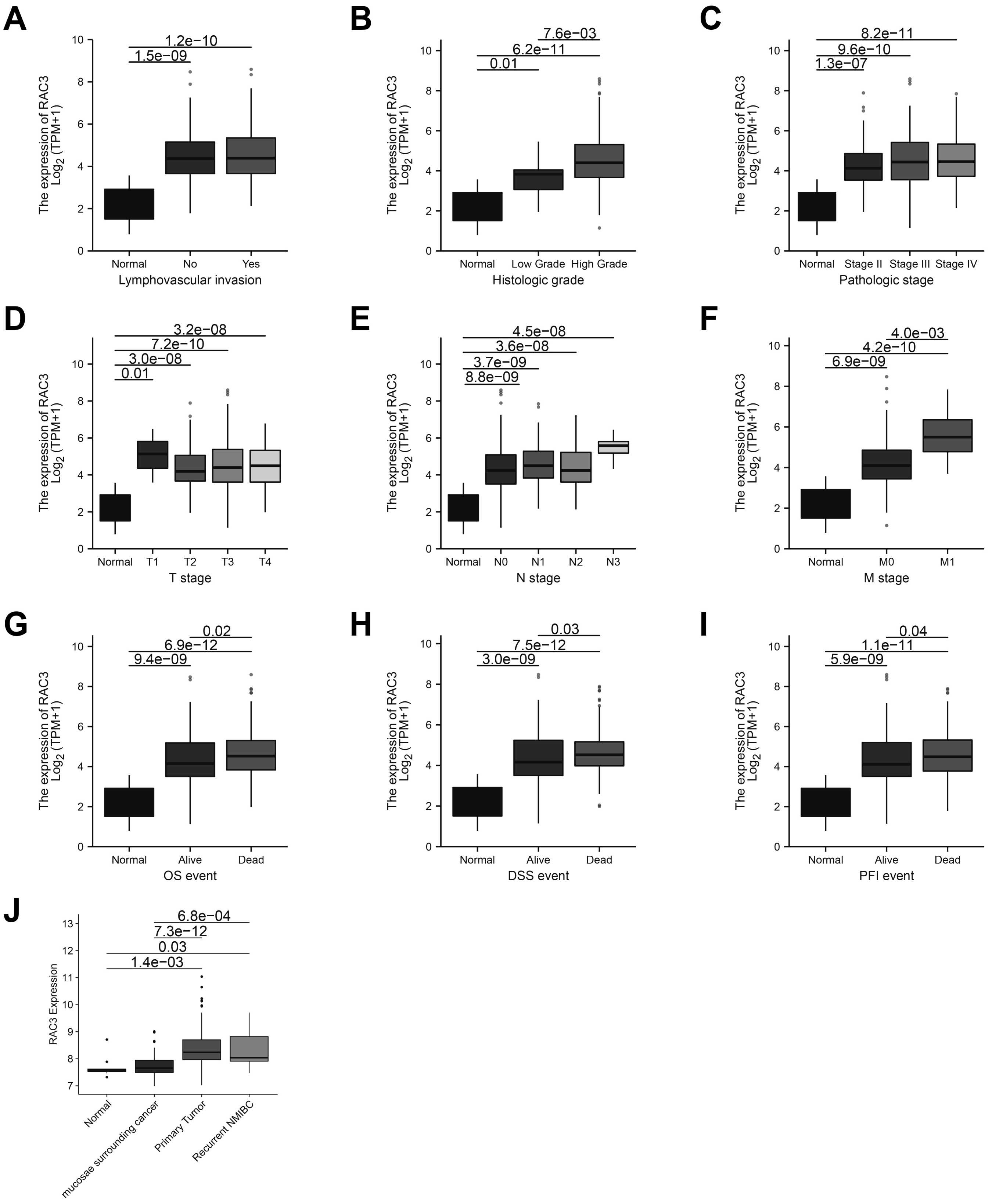

FIGURE S1 | RAC3 expression increases with cancer progression.
(A-I). RAC3 expression in different clinical subgroups of TCGA-BLCA cohort. **(J).** RAC3 expression in different clinical subgroups of GSE13507 cohort.

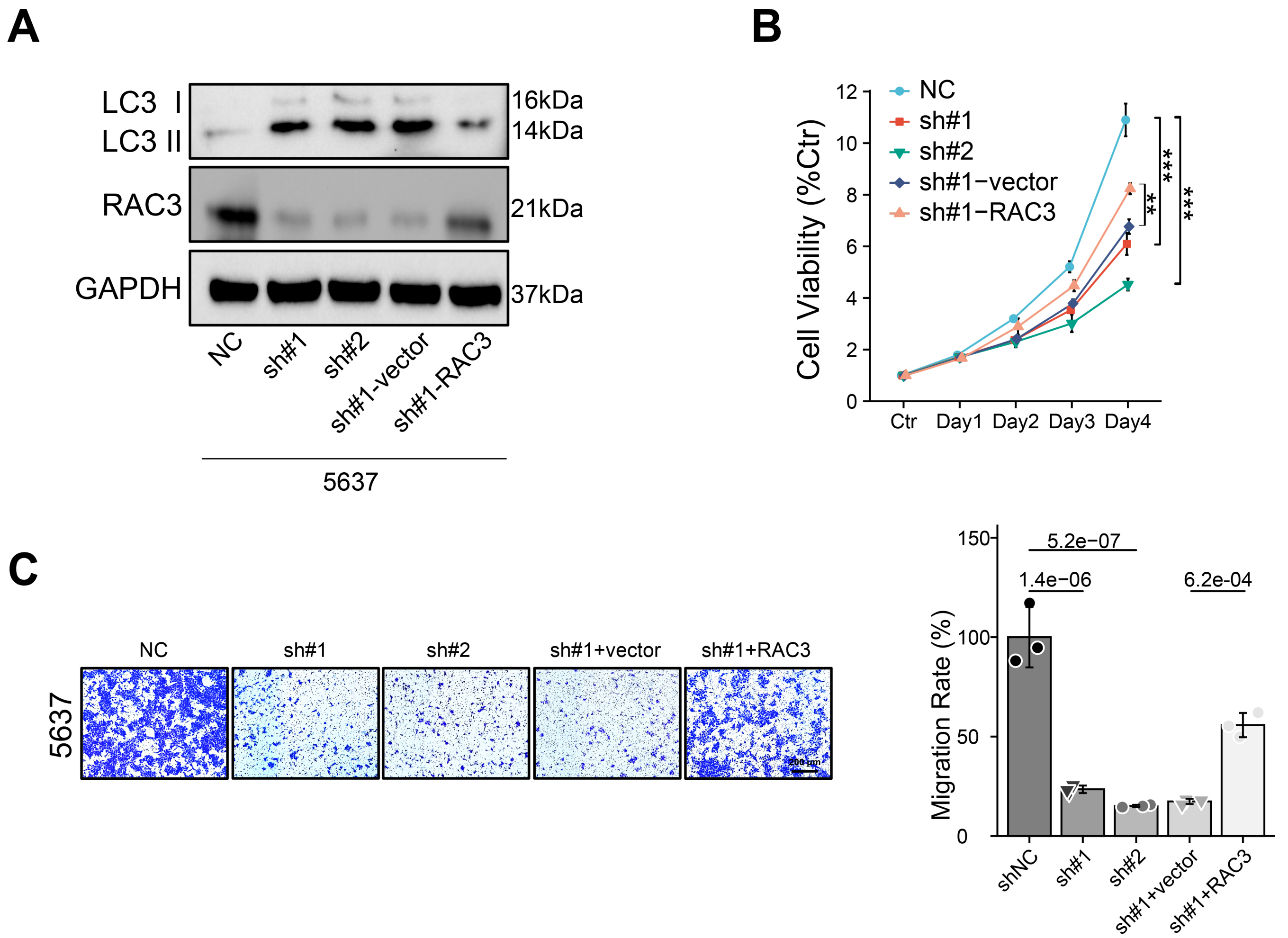

FIGURE S2 | The expression level of RAC3 affects cell proliferation, migration and autophagy. (A). Western blotting analysis of RAC3, LC3 protein expression in 5 cell lines that manipulated RAC3 expression. GAPDH was used as a loading control. **(B).** CCK8 assay comparing the cell viability as described in Figure 3. **(C).** Comparison of the migration using transwell compartments as described in Figure 4. **P<0.01, ***P<0.001.

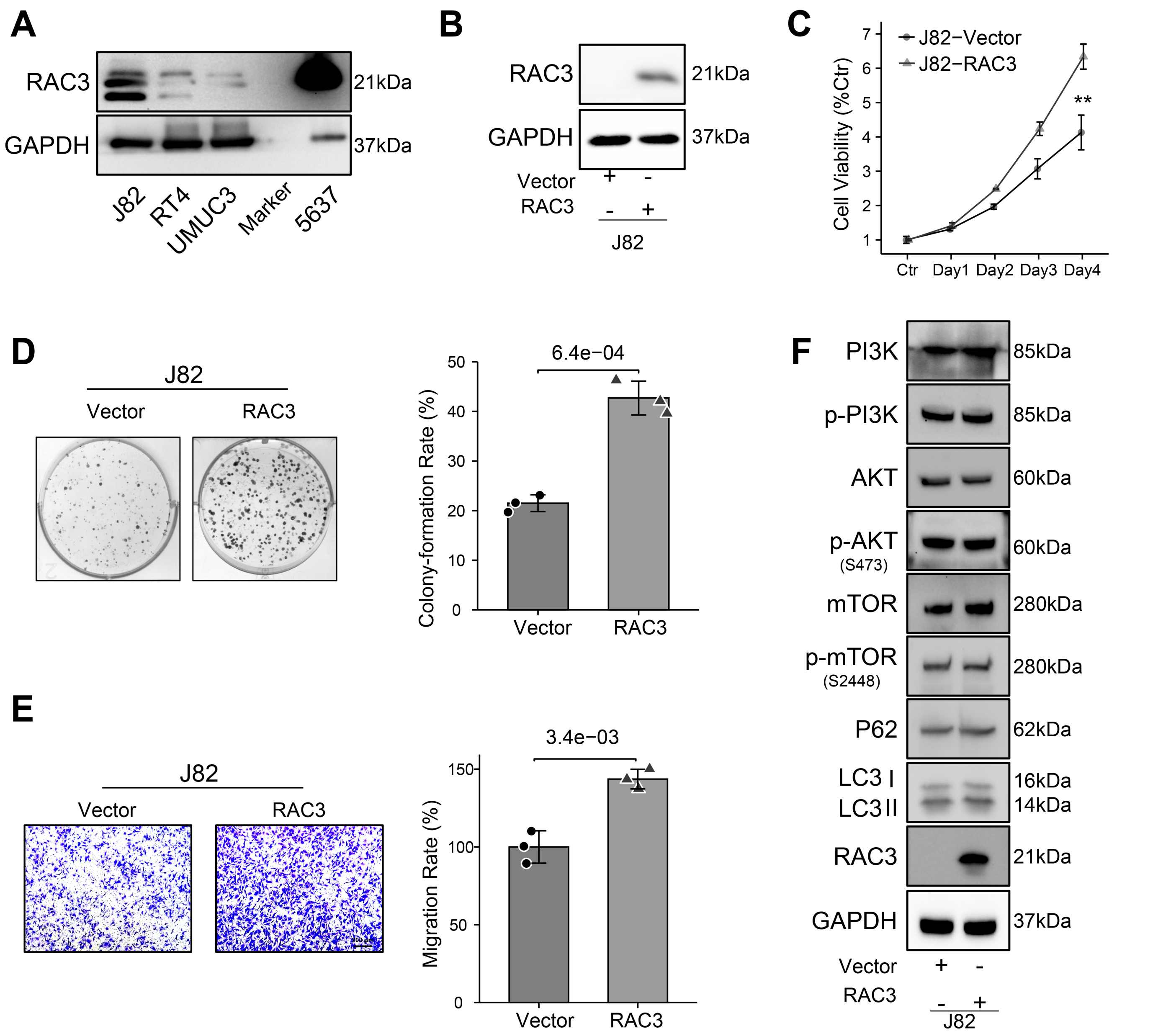

FIGURE S3 | Overexpression of RAC3 promotes proliferation and migration of BCa cells. (A-B). Western blotting analysis, 5ug of 5637 cell lysate and 40ug of J82/RT4/UMUC3 cell lysate were loaded (A), J82-vector/RAC3 cell lysate were loaded (B). GAPDH was used as a loading control. **(C)**. CCK8 assay comparing the viability as described in Figure 3. **(D)**. Colony formation assay comparing the proliferation as described in Figure 3. **(E)**. Comparison of the migration using transwell compartments as described in Figure 4. **(F)**. Western blotting analysis of the PI3K/AKT/mTOR/P62/LC3 protein expression. GAPDH was used as a loading control. **P<0.01.

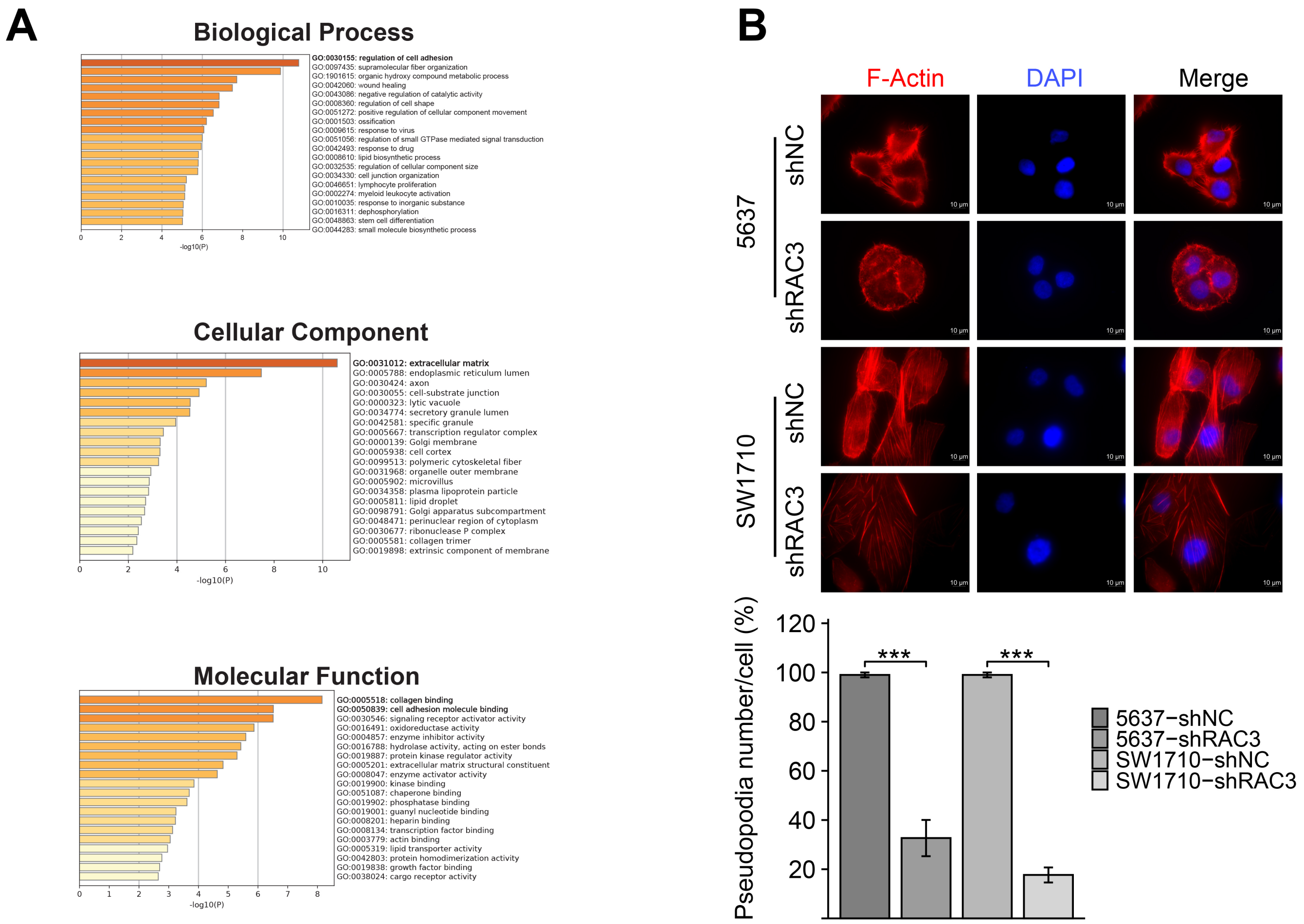

FIGURE S4 | Knockdown RAC3 attenuated the adhesion of BCa cells.
(A). GO analysis (Biological Process, Cellular Component, and Molecular Function groups) showing DEGs between 5637-shNC and 5637-shRAC3 cells were enriched in cell adhesion, extracellular matrix, collagen binding, cell adhesion molecule binding sets. **(B).** 5637-shNC/shRAC3 and SW1710-shNC/shRAC3 cells were subjected to fluorescence staining for F-actin followed by counterstaining with DAPI. The number of pseudopodia (filapodia and lamellipodia) per cell was counted by image J. ***P<0.001.

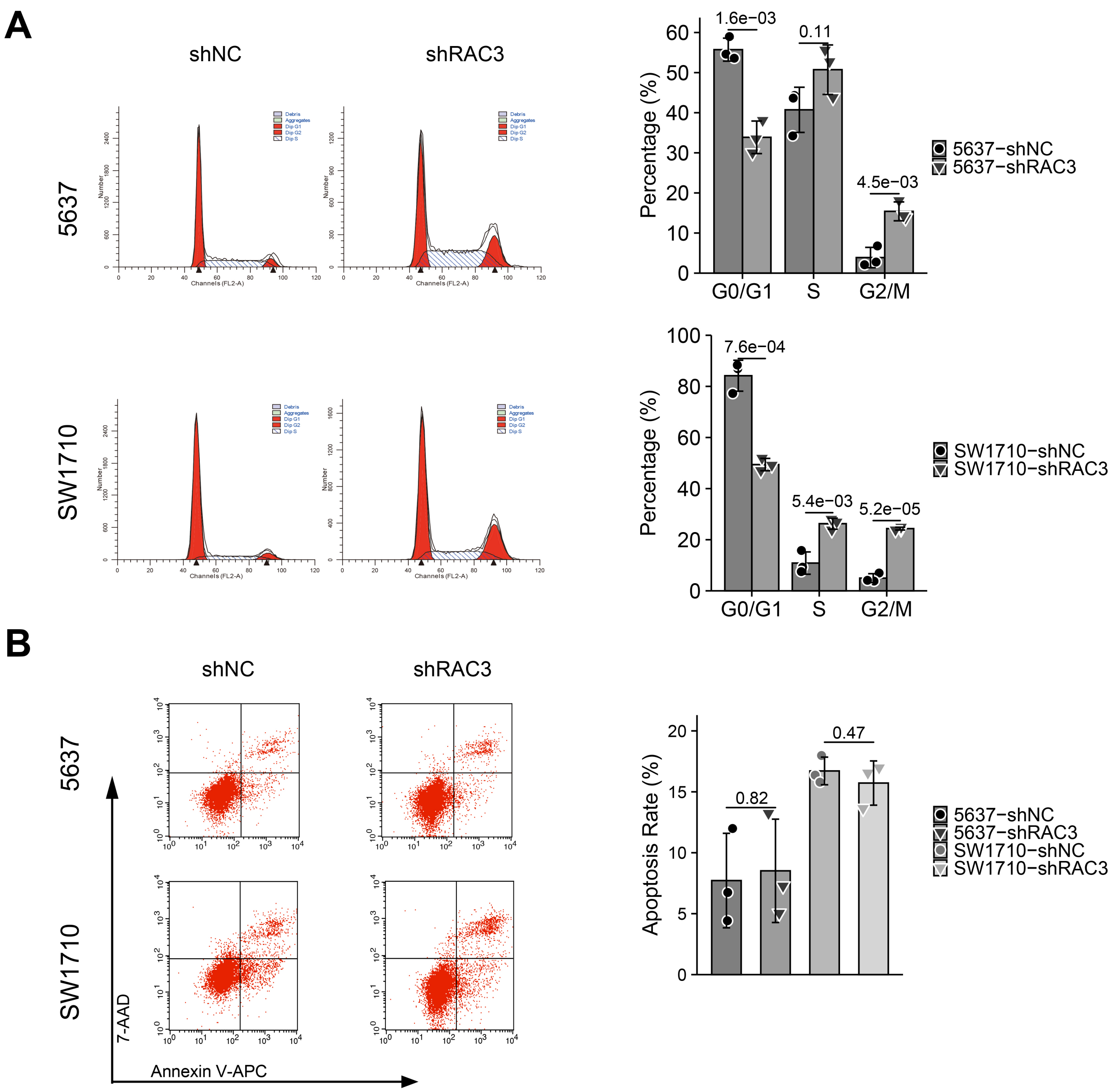

FIGURE S5 | Flow cytometry analysis.
(A). Cell cycle analysis of 5637-shNC/shRAC3 and SW1710-shNC/shRAC3 cells. **(B).** Apoptosis analysis of 5637-shNC/shRAC3 and SW1710-shNC/shRAC3 cells.

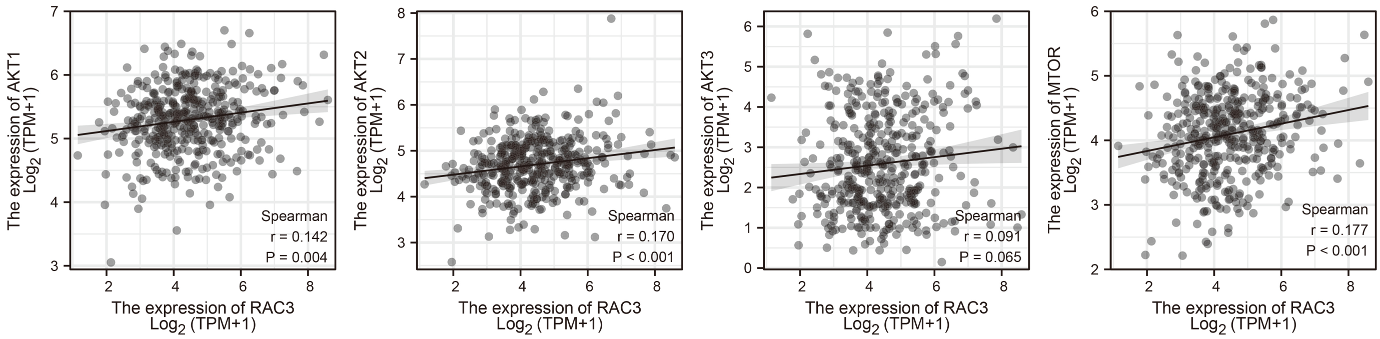

FIGURE S6 | Correlation between RAC3 and AKT/mTOR in TCGA-BLCA.
mRNA expression of RAC3 is positively correlated with AKT1, AKT2, AKT3 and mTOR. Spearman coefficient was used.

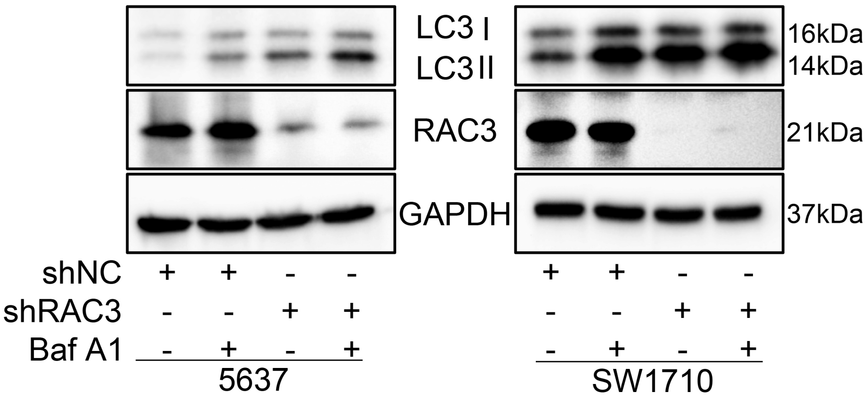

FIGURE S7 | Autophagy flux assay.

Western blotting analysis of LC3-II protein expression, shNC/shRAC3 cells were treated with bafilomycin A1 (100nM) for 24 h. GAPDH was used as a loading control.

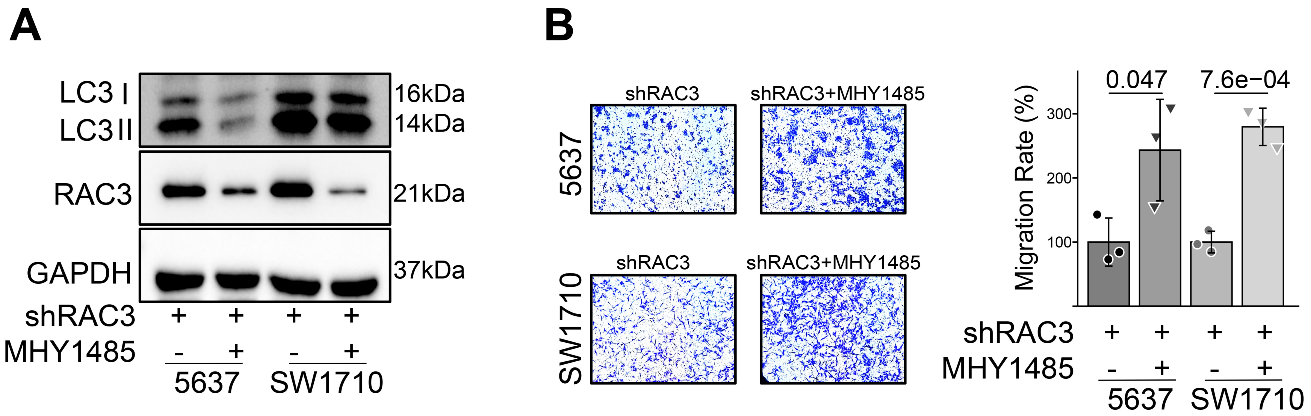

FIGURE S8 | RAC3-induced autophagic flux is mediated through the mTOR pathway. shRAC3 cells were in the presence or absence of MHY1485 (10μM) for 24h. **(A).** Western blotting analysis of RAC3, LC3 protein expression in shRAC3 cells. GAPDH was used as a loading control. **(B).** Comparison of the migration using transwell compartments as described in Figure 4.

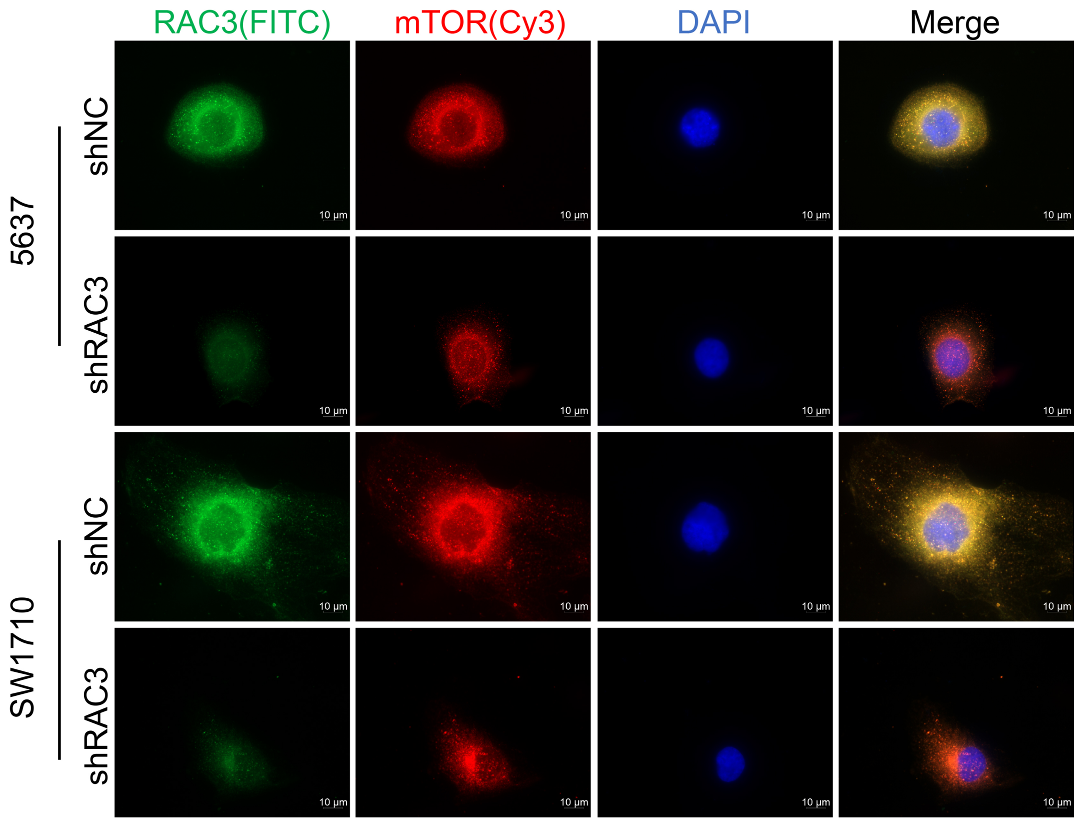

FIGURE S9 | RAC3 is colocalized with mTOR.
Cells were fixed and immunostained for RAC3 and mTOR and analyzed by immunofluorescence.

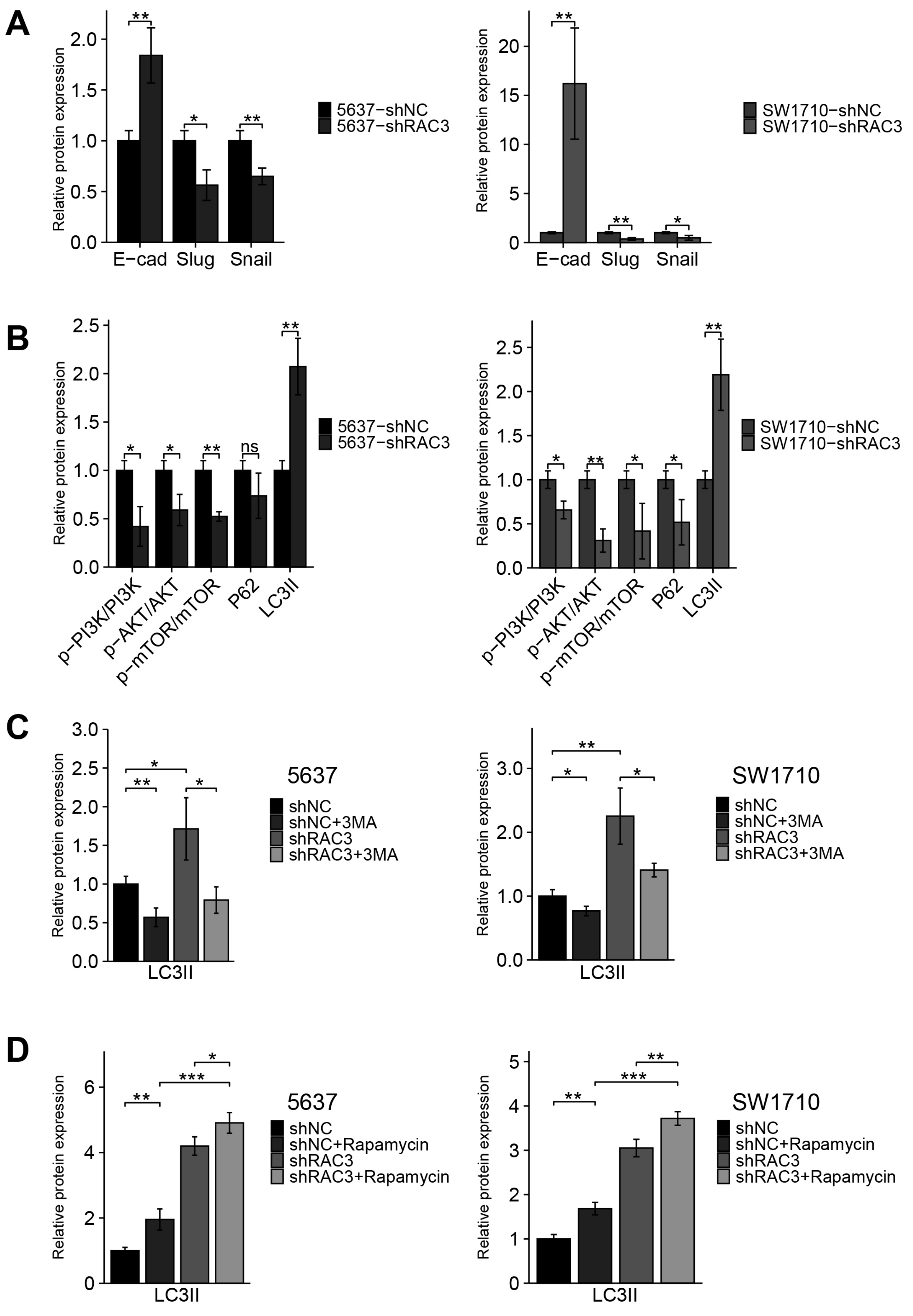

FIGURE S10 | Quantification of protein expression.

Quantification of protein expression in Figure 4I **(A)**, Figure 5BC **(B),** Figure 6A **(C)** Figure 7A **(D).** The data are presented as the mean ± S.D. from three independent experiments. *P< 0.05, **P< 0.01, ***P< 0.001.
