# Supplementary material for: RAC3 Inhibition Induces Autophagy to Impair Metastasis in Bladder Cancer Cells via the PI3K/AKT/mTOR Pathway
Source: Front Oncol. 2022 Jun 30;12:915240. doi: 10.3389/fonc.2022.915240 (PMC9279623; doi:10.3389/fonc.2022.915240)
Supplement: Supplementary file 2 [file Presentation_1.pptx]

## Slide 1
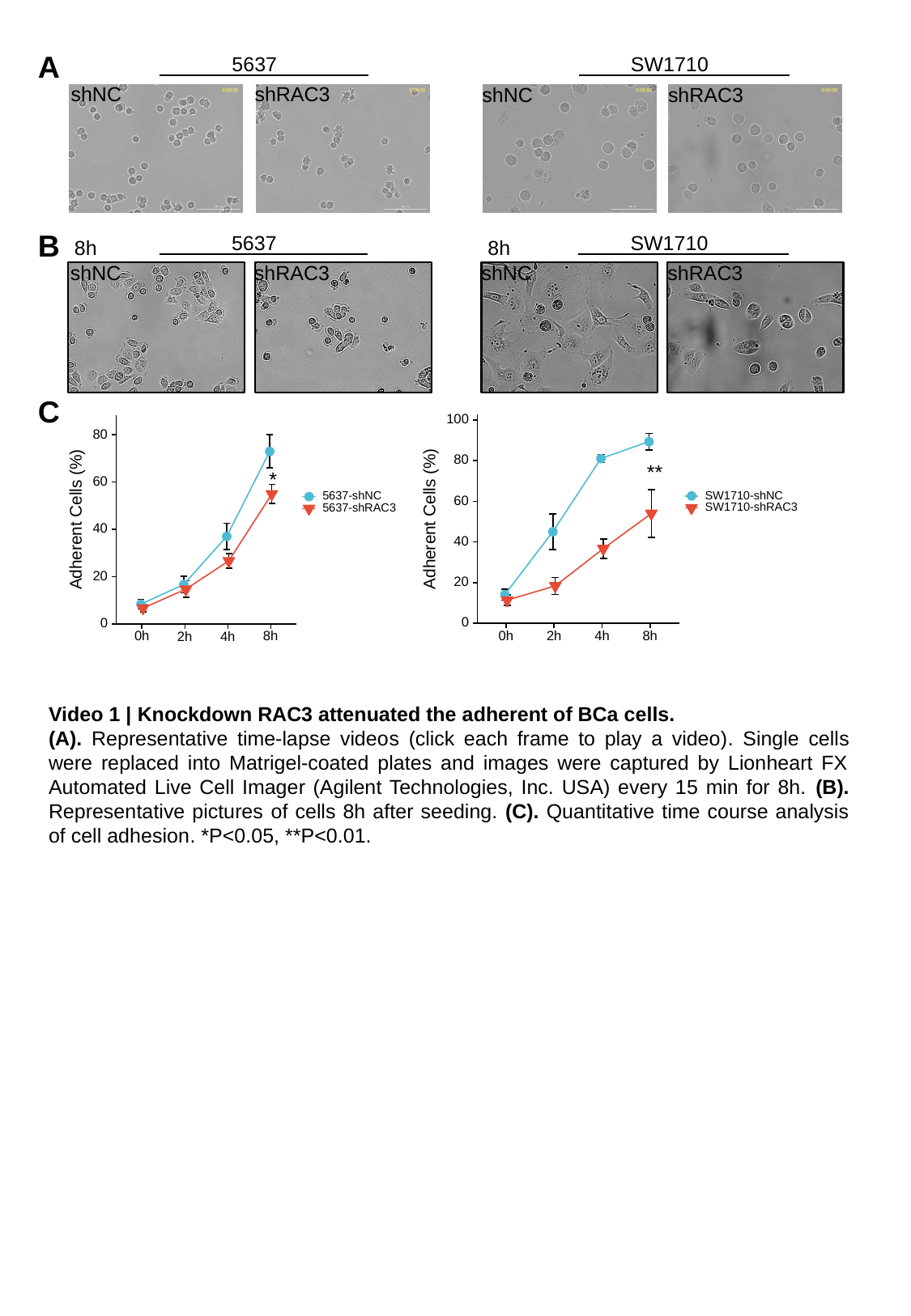

A
5637
SW1710
shNC
shRAC3
shNC
shRAC3
B
5637
SW1710
8h
8h
shNC
shRAC3
shNC
shRAC3
C
Video 1 | Knockdown RAC3 attenuated the adherent of BCa cells.
(A). Representative time-lapse videos (click each frame to play a video). Single cells were replaced into Matrigel-coated plates and images were captured by Lionheart FX Automated Live Cell Imager (Agilent Technologies, Inc. USA) every 15 min for 8h. (B). Representative pictures of cells 8h after seeding. (C). Quantitative time course analysis of cell adhesion. *P<0.05, **P<0.01.
100
80
 80
**
60
*
SW1710-shNC
5637-shNC
 60
SW1710-shRAC3
5637-shRAC3
Adherent Cells (%)
Adherent Cells (%)
40
 40
20
 20
 0
 0
0h
8h
2h
4h
0h
8h
2h
4h
